# Effect of Oxygen-Inhibited Layer of Dental Adhesives on Bond Strength: A Systematic Review

**DOI:** 10.3390/ma19010113

**Published:** 2025-12-29

**Authors:** Arpita Patangia, Lora Mishra, Manoj Kumar, Klara Saczuk, Barbara Lapinska

**Affiliations:** 1 Department of Conservative Dentistry and Endodontics, Institute of Dental Sciences, Siksha ‘O’ Anusandhan, Bhuba-neswar 751003, Odisha, India; arpita.patangia13@gmail.com; 2 Department of Periodontics and Oral Implantology, Institute of Dental Sciences and SUM Hospital, Siksha ‘O’ Anusandhan University, Bhubaneswar 751003, Odisha, India; manojkumar@soa.ac.in; 3 Department of General Dentistry, Medical University of Lodz, 251 Pomorska St., 92-213 Lodz, Poland; klara.saczuk@umed.lodz.pl

**Keywords:** oxygen inhibition layer, bonding agent, bond strength

## Abstract

The aim of the study was to evaluate the effect of the oxygen-inhibited layer on the bond strength of dental adhesives. The protocol was registered in PROSPERO. PRISMA 2020 guidelines were followed. The focused structured question using Population (P), Intervention (I), Comparison (C), and Outcome (O) was: “What is the effect of oxygen inhibited layer on bonding strength of dental adhesives?” The literature was screened via PubMed, Google Scholar, Scopus, and Web of Science. The last search was carried out in September 2024 with an English language restriction. Two reviewers independently performed screening and evaluation of articles. A total of 71 articles were retrieved from databases, in which only 35 articles were selected for full-text analyses. After implementing the exclusion criteria, eight studies were evaluated and included in the review. The results showed that the presence of an oxygen-inhibited layer led to an increased bond strength when light-cured composite resin was used, but there was a decrease in bond strength and an increased bond failure rate when chemically cured composite was used upon dental adhesive application. Meta-analysis could not be performed due to heterogeneity in the studies. The presence of an oxygen-inhibited layer is beneficial in improving the interfacial bond strength when used with light-cured composite resin (when light curing was performed in accordance with the manufacturer’s instructions).

## 1. Introduction

Dental adhesives play a critical role in contemporary restorative dentistry by forming the interface between restorative materials and tooth substrates. The durability and effectiveness of this adhesive interface largely determine the longevity and clinical success of restorations. Among the multiple factors influencing adhesive performance, the formation of the oxygen-inhibited layer (OIL) during polymerization has received increasing attention. This layer consists of unpolymerized, or partially polymerized resin monomers formed due to inhibition of free-radical polymerization by atmospheric oxygen [1,2]. While the presence of OIL is known to facilitate copolymerization with subsequently placed resin layers, its influence on bond strength with enamel and dentin remains controversial.

The oxygen-inhibited layer is characterized as a thin, sticky surface layer that develops when oxygen interferes with resin polymerization, resulting in a reduction in the availability of active photoinitiators [2,3]. The thickness of this layer plays a crucial role in interfacial bonding; a thin OIL may promote interdiffusion and chemical bonding between composite layers, whereas a thicker layer can compromise interfacial integrity and reduce bond strength [4,5]. Reported thickness values of the oxygen-inhibited layer in composite resins range from approximately 4 µm to 40 µm, depending on several factors, including monomer composition, initiator–activator systems, filler morphology, concentration of free radicals, and oxygen diffusion dynamics [6].

Monomer composition has a pronounced influence on oxygen inhibition. Adhesives with higher concentrations of low-viscosity methacrylate monomers such as HEMA tend to exhibit thicker oxygen-inhibited layers, consistent with observations that decreased resin viscosity facilitates oxygen diffusion [7,8]. In addition, filler particles may act as physical barriers or pathways for oxygen penetration, thereby modulating OIL thickness [9]. Surface free energy, which represents the combined effect of dispersion forces, hydrogen bonding, and polar interactions, is also influenced by the presence of the oxygen-inhibited layer and may further affect adhesive wettability and bonding performance [9]. Fatigue resistance of the bonded interface, an important determinant of long-term clinical durability, has likewise been associated with the quality of the adhesive interface formed in the presence or absence of an OIL [3].

Various strategies have been proposed to reduce or modify the oxygen-inhibited layer. Thermal activation at temperatures between 110 °C and 120 °C has been shown to decrease OIL thickness without altering resin composition, potentially by increasing the availability of active radicals [9]. The incorporation of phosphine derivatives such as triphenylphosphine (TPP) or 4-(diphenylphosphino)styrene (DPPS) into photoinitiator systems has also been reported to enhance polymerization efficiency under aerobic conditions. While TPP raises toxicological concerns, DPPS is considered suitable for dental applications and has been shown to limit oxygen-related inhibition without compromising color stability [10].

**Figure 1:**
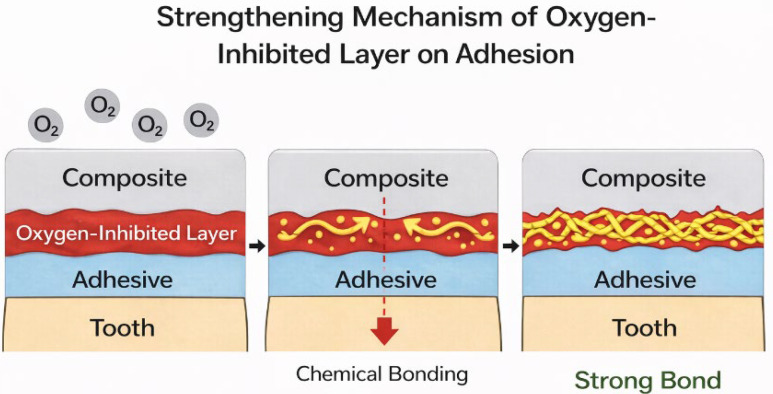
A diagram of the mechanism of action of oxygen inhibition on bond strength.

The influence of the oxygen-inhibited layer on bond strength remains inconsistent across the available literature (Figure 1). Some studies have reported improved bond strength in the presence of OIL, particularly when self-etch adhesive systems are used, whereas others have found minimal or no effect [5,11,12]. Experimental comparisons of composites cured under air versus nitrogen atmospheres have further suggested that surface inhibition may not significantly affect incremental bond strength under certain conditions [5,11,12]. Post-polymerization finishing and polishing procedures have been shown to partially remove the oxygen-inhibited layer, thereby improving color stability and wear resistance by eliminating the superficial layer of unpolymerized resin [6]. Additional barrier techniques, such as the use of Mylar strips or thermal activation, have also been proposed to limit oxygen exposure during polymerization [9,13,14]. Ethanol application has been reported as an effective method for removing the oxygen-inhibited layer; however, its use requires caution to avoid unintended effects on resin structure or the adhesive interface [15].

Despite the extensive investigation of dental adhesives, the oxygen-inhibited layer is often discussed as a secondary phenomenon rather than as an independent interfacial variable influencing adhesive performance. The available evidence regarding its role in modulating bond strength, surface free energy, interfacial characteristics, and failure patterns remains fragmented and inconclusive. The present systematic review was therefore conducted to provide a focused synthesis of available in vitro evidence evaluating the oxygen-inhibited layer as a primary determinant of adhesive bond performance. By distinguishing the differential effects of the oxygen-inhibited layer in conjunction with light-cured and chemically cured resin composites, this review aims to address a previously underexplored aspect of adhesive dentistry with potential implications for the long-term performance and prognosis of dental restorations.

## 2. Materials and Methods

The protocol for this systematic review is registered with PROSPERO under registration number CRD42024574190. The review was carried out following the Preferred Reporting Items for Systematic Reviews and Meta-Analyses (PRISMA) statement guidelines [16] as detailed in the PRISMA 2020 Checklist (Table S1).

### 2.1. Study Selection

The research question was established as “What is the effect of oxygen inhibited layer on bonding strength of dental adhesives?”. Then the PICO framework was implemented:

- Population (P): Studies involving human or bovine teeth undergoing bonding protocol for composite restoration;
- Intervention (I): Bonding protocol for enamel and dentin;
- Comparison (C): Oxygen inhibition layer strategies;
- Outcome (O): shear bond strength, micro-tensile bond strength, SEM analysis, surface free energy.

The primary goal was to evaluate the bond strength of enamel, dentin, and composite substrate. The secondary goal was to evaluate marginal integrity, microleakage, and failure mode of dental restorations.

The eligibility criteria are presented in Table 1.

### 2.2. Literature Search

In line with PRISMA guidelines, PubMed, Scopus, Web of Science, and Google Scholar was searched for English, open-access articles published between January 2000 and August 2024. Search strategies were tailored for each database, and reference lists were reviewed to ensure comprehensive coverage.

The search strategy included the following keywords: (“oxygen inhibition layer” OR “oxygen inhibited layer” OR “oxygen inhibited photopolymerization” OR “uncured resin” OR “oxygen-inhibition layer”) AND (“effect” OR “impact” OR “influence” OR “affect” OR “result”) AND (“bonding agent” OR “dba” OR “adhesive system” OR “dental adhesion” OR “dental adhesive” OR “adhesive systems” OR “adhesive” OR “dental adhesives” OR “bonding agents” OR “bond” OR “bond system”) AND (“bond strength” OR “shear bond strength” OR “micro-tensile bond strength” OR “surface energy” OR “surface discoloration” OR “water contact angle” OR “fracture strength”.

Records retrieved from the database search were imported into Rayyan (Boston). Duplicate entries were removed by LM and AP, and titles and abstracts were screened against the inclusion criteria. Studies meeting the criteria underwent full-text review, with only open access articles included in the final analysis.

### 2.3. Data Extraction

Data were extracted using a standardized form and tabulated in Excel (Microsoft Office 2019). Extracted information included bibliographic details (journal, title, authors, year), study design, sample preparation methods, adhesives used, approaches to OIL reduction, bond strength testing methods, OIL thickness, surface energy, and fatigue strength [10,17].

### 2.4. Quality Assessment

The QUIN (Quality Assessment tool) was used to evaluate the quality and risk of bias for the included studies (tool for assessing in vitro studies) [18]. The QUIN tool included 12 points along with the scoring and grading criteria to evaluate the content quality and reliability. Each of the criteria was scored as adequately specified (2 points), inadequately specified (1 point), or not specified (0 points), and if not applicable, was excluded from the study. The scores obtained were then graded as high risk (<50%), medium risk (50 to 70%), and low risk (>70%). The final score was calculated as (total score × 100)/(2 × number of applicable criteria).

## 3. Results

**Figure 2:**
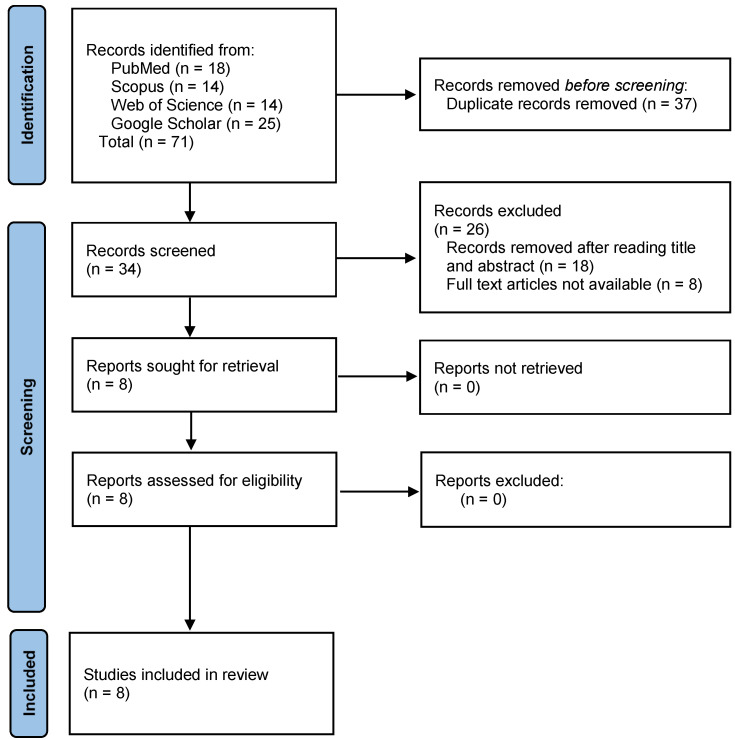
PRISMA flow diagram.

The initial stage of the literature review involved a comprehensive search across four widely recognized databases: PubMed, Scopus, Web of Science, and Google Scholar. This extensive search yielded 71 articles (Figure 2). The next step involved identifying and removing duplicate entries among the retrieved records, and it yielded 34 articles. The remaining articles underwent a detailed screening process, starting with a thorough review of their titles and abstracts. During this phase, 18 articles were found to be unrelated to the study’s focus and were excluded (Table S2). Full-text versions of the articles were only further included, with 8 articles being excluded at this stage. As a result of this systematic and methodical filtering process, a final total of eight articles met all the predefined eligibility criteria. These articles were deemed both relevant to the study’s objectives and accessible for detailed analysis.

### 3.1. Sample Size and Preparation

A total of 730 non-carious, intact teeth were examined, consisting of 450 bovine teeth and 280 human teeth. The specimens primarily included human molars and bovine incisors from both the maxillary and mandibular arches. Each tooth was thoroughly cleaned and sectioned mesiodistally to prepare the samples. The coronal portions of the teeth were retained, with the root portions removed to focus on the desired anatomical structures [5,11,19,20,21,22,23,24]. Depending on the specific requirements of each study, either the enamel or dentin surface of the tooth was exposed by sectioning. In most studies, this process was performed using a slow-speed handpiece. The prepared tooth samples were then embedded in acrylic studs to facilitate handling and further preparation. The exposed surfaces were ground flat using sandpapers with progressively finer grit sizes, including 180, 320, 600, 1200, and 1400 grit [5,11,19,20,21,22,23,24]. In one study, an automatic polishing system was used for surface preparation [22]. After grinding and polishing, the prepared surfaces were thoroughly washed and dried using oil-free compressed air to ensure cleanliness and uniformity before further experimental procedures [22].

### 3.2. Overview of Studies

Eight in vitro studies met the inclusion criteria and were included in the qualitative synthesis. The studies demonstrated a considerable variability in experimental design, including differences in composite curing mechanism, oxygen-inhibited layer (OIL) modification, and outcome assessment. An overview of study characteristics is presented in Table 2 and Table 3.

### 3.3. Bond Strength Outcomes According to Oxygen-Inhibited Layer Condition

All included studies evaluated bond strength as a primary goal. When results were synthesized in terms of the composite curing mechanism, a consistent pattern emerged. In studies using light-cured resin composites, preservation of the oxygen-inhibited layer was generally associated with comparable or higher bond strength values compared with its removal. In contrast, studies involving chemically cured composites consistently reported reduced bond strength in the presence of the oxygen-inhibited layer.

These findings were observed across different testing methods, including shear bond strength and microtensile bond strength, and were independent of specific adhesive brands. A number of models have been used for testing shear bond strength, including Electroplus E1000 [19], Instron 4466 [22], Instron 4204 [5,11,20], Instron 5500 [23,24], and micro-tensile bond tester [21]. Quantitative outcomes supporting these trends are summarized in Table 4.

### 3.4. Influence of Etching Mode and Oxygen-Inhibited Layer Modification

The effect of the oxygen-inhibited layer on bond strength was further influenced by etching strategy (Table 4). Studies that used self-etch and total-etch approaches showed that the change of direction of bond strength remained primarily dependent on the composite curing mechanism rather than etching mode alone (Table 4). Methods used to modify or remove the oxygen-inhibited layer (including ethanol application, extended curing time, and physical barriers) demonstrated variable effects, with no single approach consistently improving bond strength across all study conditions.

### 3.5. Surface Free Energy and Wettability

Surface free energy was evaluated in a subset of studies [5,11,20,23,24] (Table 3). Removal of the oxygen-inhibited layer was frequently associated with increased surface free energy [5,11,23]; however, this did not consistently translate into higher bond strength values. Conversely, one study [24] reported reduced surface free energy in the presence of the oxygen-inhibited layer alongside improved bonding performance. These inconsistent findings indicate that surface free energy alone cannot explain the observed bond strength outcomes.

### 3.6. Failure Mode Analysis

Failure mode analysis was reported in most of the included studies (Table 3). Adhesive failure predominated regardless of oxygen-inhibited layer condition, indicating that the adhesive interface remained the primary site of failure. Although some studies reported mixed or cohesive failures under specific conditions, variations in classification and reporting limited comparative interpretation. No consistent failure mode pattern could be attributed solely to the presence or absence of the oxygen-inhibited layer.

### 3.7. Quality Assessment of Included Studies

According to the QUIN tool, most studies demonstrated a medium risk of bias, with one study classified as high risk. Common methodological shortcomings included lack of sample size calculation, absence of blinding, limited reporting of randomization, and insufficient operator standardization. These limitations reduce confidence in the magnitude and consistency of reported effects. However, the risk of bias score ranged from 40% to 70% across the included studies (Table 5).

### 3.8. Meta-Analysis

Due to substantial heterogeneity in the methodologies and reported outcomes of the included studies, a meta-analysis was not feasible for this systematic review. The observed variations across studies precluded meaningful pooling of data for quantitative analysis. This heterogeneity encompassed differences in study design, materials used, experimental conditions, and outcome measures, making it statistically insufficient to combine results. Instead, a structured descriptive synthesis was made. Feasible results were grouped and interpreted according to predefined subgroups, including composite curing mechanism, etching mode, and presence or absence of the oxygen-inhibited layer.

## 4. Discussion

The present systematic review synthesized the available in vitro evidence regarding the influence of the oxygen-inhibited layer (OIL) on the bond strength of dental adhesives to tooth substrates and composite restorations. Only a limited number of studies have addressed this topic, with eight relevant investigations identified. The findings indicate that bond strength outcomes are influenced by multiple interacting variables, including adhesive strategy, composite curing mechanism, tooth substrate, and the method used to modify or remove the oxygen-inhibited layer. However, given the heterogeneity of study designs and the moderate methodological quality of the included evidence, these findings should be interpreted cautiously.

### 4.1. Effect of the Presence or Absence of the Oxygen-Inhibited Layer

The thickness and characteristics of the oxygen-inhibited layer are known to be influenced by factors such as monomer composition, solvent system, initiator type, adhesive viscosity, composite formulation, and curing parameters. Several experimental approaches, including ethanol application, water spray, and use of inert barriers, have been used to modify or remove the OIL.

Across the included studies, preservation of the oxygen-inhibited layer was frequently associated with higher initial bond strength values when light-cured resin composites were used [19]. This observation suggests that the presence of an OIL may facilitate interfacial interaction between the adhesive and the overlying composite, potentially through enhanced copolymerization or interdiffusion. However, these findings were not uniformly reported across all studies; most evidence relates to short-term bond strength testing. Therefore, the apparent beneficial effect of the OIL should not be generalized beyond the specific experimental conditions evaluated.

In contrast, when self-etch adhesives were combined with chemically cured resin composites, reduced bond strength was consistently reported in the presence of the oxygen-inhibited layer [11]. This effect has been attributed to interactions between residual acidic monomers and peroxide–amine initiator systems, which may interfere with polymerization. While this explanation is mechanistically plausible, it is supported by a limited number of studies and should be regarded as hypothesis-generating rather than definitive.

Overall, the available evidence indicates that the effect of the oxygen-inhibited layer on bond strength is dependent on the curing mechanism of the composite resin, underscoring the need for further studies using standardized experimental protocols.

### 4.2. Effect of Increasing Curing Time

The formation of the oxygen-inhibited layer is primarily governed by oxygen diffusion into the uncured resin; however, curing parameters such as exposure time and light energy may also influence its thickness. Increasing curing time has been reported to reduce the thickness of the OIL by promoting further polymerization of residual monomers.

One included study suggested that extended curing time was associated with a reduction in bond strength and increased bond failure [22]. This observation may be related to excessive polymerization within the inhibited layer, potentially reducing its capacity to contribute to chemical bonding at the interface. Nevertheless, evidence supporting this effect is limited, and variations in curing protocols across studies prevented direct comparison. Consequently, no definitive conclusions regarding optimal curing time in relation to oxygen inhibition can be drawn from the current evidence.

### 4.3. Effect of Substrate Evaluated

Although several studies evaluated adhesive bond strength to either enamel or dentin, none directly compared the effect of the oxygen-inhibited layer on these substrates under identical experimental conditions. Given the compositional and structural differences between enamel and dentin, substrate-specific variations in bonding behavior are plausible. However, the available evidence does not permit conclusions regarding differential substrate effects of the oxygen-inhibited layer, highlighting a gap in the current literature.

### 4.4. Effect of Surface Free Energy

Surface free energy is a key determinant of adhesive wettability and interfacial interaction. The oxygen-inhibited layer has been reported to alter surface characteristics, potentially affecting the balance between Lewis acid and Lewis base components and, consequently, bonding behavior.

Some studies included in this review reported reduced surface free energy in the presence of the oxygen-inhibited layer, accompanied by increased bond strength, whereas others observed increased surface free energy under similar conditions [23]. These conflicting findings suggest that the relationship between surface free energy, oxygen inhibition, and bond strength is complex and likely influenced by additional variables such as adhesive composition and substrate characteristics. Based on the current evidence, surface free energy alone cannot be considered a reliable predictor of bond strength outcomes in relation to the oxygen-inhibited layer.

### 4.5. Overall Interpretation

Taken together, the findings of this systematic review suggest that the oxygen-inhibited layer may influence interfacial characteristics and bond strength under certain experimental conditions. However, the limited number of studies, heterogeneity of methodologies, and moderate risk of bias preclude definitive conclusions. The observed trends should therefore be considered indicative rather than confirmatory, and further well-designed, standardized in vitro studies are required to clarify the role of the oxygen-inhibited layer in adhesive dentistry.

### 4.6. Limitations and Future Directions

The limitations of this systematic review involve mainly the variations in the quality of included studies, publication bias, and the need for further research to determine the clinical relevance of these findings. Future studies should aim to determine the relation between surface free energy and oxygen-inhibited layer, the effect of the oxygen-inhibited layer and bond strength on enamel and dentin, and a comparison between light-cured and chemically cured composites on OIL of the modified adhesive layer.

The findings of this systematic review should be interpreted while being aware of its limitations. The included studies exhibited substantial methodological and clinical heterogeneity with respect to substrate type, adhesive strategy, composite curing mechanism, and methods used to modify the oxygen-inhibited layer. In addition, most studies demonstrated moderate to high risk of bias, primarily due to lack of sample size calculation, absence of blinding, and incomplete reporting of randomization procedures. Because of this heterogeneity, quantitative meta-analysis was not feasible. Although subgroup analyses were predefined and explored using a structured descriptive approach, the limited number of studies within each subgroup precluded extensive comparative inference. Consequently, the observed patterns—particularly the differential effect of the oxygen-inhibited layer on light-cured versus chemically cured composites—should be regarded as hypothesis-generating rather than definitive.

The oxygen-inhibited layer plays a major role in determining the bond strength, bond failure, and resistance to shrinkage among different composite restorations and should be considered when evaluating a long-term prognosis and outcome of a restoration. Further well-designed studies are needed to explore these interactions and effects fully. This research is crucial for refining optimal restorative protocols and ultimately improving clinical outcomes in restorative dentistry.

## 5. Conclusions

Within the limitations of the available in vitro evidence, this systematic review suggests that the oxygen-inhibited layer may influence the bond strength of dental adhesives under specific experimental conditions. The effect appears to vary according to the curing mechanism of the resin composite, with preservation of the oxygen-inhibited layer generally associated with comparable or higher bond strength when light-cured composites are used, and reduced bond strength observed with chemically cured composites.

However, the limited number of included studies, substantial methodological heterogeneity, and moderate to high risk of bias restrict the strength of these conclusions. As a result, the observed trends should be interpreted cautiously and should not be directly extrapolated to clinical performance. Current evidence is insufficient to support definitive recommendations regarding routine modification or removal of the oxygen-inhibited layer in clinical practice.

Further well-designed, standardized in vitro studies are required to clarify the role of the oxygen-inhibited layer across different adhesive strategies, substrates, and composite curing mechanisms, and to determine its potential relevance to long-term restorative outcomes.

## Figures and Tables

**Table 1:** Eligibility criteria.

Inclusion Criteria	Exclusion Criteria
Studies investigating the effect of the presence or absence of an oxygen-inhibiting layer of dental adhesives on bond strength.	Review articles, letters to the editor, clinical studies, and case reports.
In vitro studies	Studies not related to dental adhesives and oxygen-inhibited layers.
Studies published in the English language.	Articles not particularly related to the research topic.
Articles with open access.	
Articles published from January 2000 to August 2024.	
Studies conducted on extracted non-carious and intact human and bovine teeth specimens.	

**Table 2:** Characteristics of included studies.

Author (Year)	Substrate	Adhesive Strategy	Composite Type	OIL Modification Method	Primary Outcome
Kim et al. (2006) [22]	Human dentin	Etch-and-rinse	Light-cured	Increased curing time/Mylar strip	μTBS
Koga et al. (2011) [5]	Bovine dentin	One-step self-etch	Light-cured	Ethanol	SBS
Oyama et al. (2012) [23]	Bovine enamel	One-step self-etch	Light-cured	Ethanol	SBS
Yamaji et al. (2013) [11]	Bovine dentin	One-step self-etch	Chemical-cured	Ethanol	SBS
Yamaji et al. (2014) [20]	Bovine dentin	Two-step self-etch	Chemical-cured	Ethanol	SBS
Ueta et al. (2016) [24]	Bovine enamel	Multi-strategy	Light-cured	Ethanol	SBS
Ouchi et al. (2017) [19]	Human enamel	Universal adhesive	Light-cured	Ethanol	Fatigue strength
Mikhlin et al. (2019) [21]	Glass slide	Etch-and-rinse/self-etch	Light-cured	Flowable layer	μTBS

SBS = shear bond strength; μTBS = microtensile bond strength; OIL = oxygen-inhibited layer.

**Table 3:** Tests performed in the included studies.

Study (Year)	Bond Strength Test	Fatigue Test	Surface Free Energy/Contact Angle	Degree of Conversion	OIL Thickness Measurement	Failure Mode Analysis
Kim et al. (2006) [22]	μTBS	—	—	—	✓	✓
Koga et al. (2011) [5]	SBS	—	✓	—	—	✓
Oyama et al. (2012) [23]	SBS	—	✓	—	—	✓
Yamaji et al. (2013) [11]	SBS	—	✓	—	—	✓
Yamaji et al. (2014) [20]	SBS	—	✓	—	—	✓
Ueta et al. (2016) [24]	SBS	—	✓	—	—	✓
Ouchi et al. (2017) [19]	SBS	✓	—	—	—	✓
Mikhlin et al. (2019) [21]	μTBS	—	—	✓	—	—

SBS = shear bond strength; μTBS = microtensile bond strength; OIL = oxygen-inhibited layer; ✓ = test performed.

**Table 4:** Data extracted from included studies: Oxygen-inhibited layer and bond strength.

Study (Year)	Composite Curing Mode	Etching Mode	OIL Condition	Bond Strength (MPa)	Fatigue Strength (MPa)	Surface Free Energy (mN/m)
Kim et al. (2006) [22]	Light-cured	Total-etch (H_3_PO_4_)	Present	37.4–57.4	Not reported	Not assessed
			Absent	35.1–36.7		
Koga et al. (2011) [5]	Light-cured	Self-etch	Present	17.5–18.4	Not reported	35.5–37.6
			Absent	13.2–13.6		41.2–46.5
Oyama et al. (2012) [23]	Light-cured	Self-etch	Present	15.8–17.7	Not reported	37.4–38.0
			Absent	12.8–14.4		43.1–43.7
Yamaji et al. (2013) [11]	Chemical-cured	Self-etch	Present	4.8–5.2	Not reported	35.4–38.1
			Absent	7.6–8.0		44.2–46.5
Yamaji et al. (2014) [20]	Chemical-cured	Self-etch	Present	9.7–11.8	Not reported	41.1–42.6
			Absent	11.3–12.5		47.5–50.0
Ueta et al. (2016) [24]	Light-cured	Total-etch/self-etch	Present	25.4–38.8	Not reported	60.1–64.4
			Absent	19.8–35.8		52.4–55.2
Ouchi et al. (2017) [19]	Light-cured	Pre-etch/no pre-etch	Present	40.6–44.3	20.3–22.2	Not assessed
			Absent	34.1–36.6	16.0–18.1	
Mikhlin et al. (2019) [21]	Light-cured	Total-etch/self-etch	Present	18.8–39.6	Not reported	Not assessed
			Absent	11.9–34.8		

**Table 5:** Quality assessment of included studies.

Study (Year)	Overall QUIN Score (%)	Risk of Bias Category	Key Methodological Limitations
Kim et al. (2006) [22]	66.6	Moderate	No sample size calculation; no blinding
Koga et al. (2011) [5]	52.0	Moderate	No randomization; no blinding
Oyama et al. (2012) [23]	52.0	Moderate	No randomization; limited reporting
Yamaji et al. (2013) [11]	52.0	Moderate	No blinding; no sample size calculation
Yamaji et al. (2014) [20]	58.3	Moderate	No blinding; limited operator details
Ueta et al. (2016) [24]	52.0	Moderate	No randomization; no blinding
Ouchi et al. (2017) [19]	62.5	Moderate	Partial randomization; no blinding
Mikhlin et al. (2019) [21]	45.8	High	Poor reporting; no randomization or blinding

## Data Availability

No new data were created or analyzed in this study. Data sharing is not applicable to this article.

## Supplementary Materials

The following supporting information can be downloaded at: https://www.mdpi.com/article/10.3390/ma19010113/s1, Table S1. PRISMA 2020 Checklist; Table S2: The list of the excluded articles with reasons for exclusion. Refs. [25,26,27,28,29,30,31,32,33,34,35,36,37,38,39,40,41,42,43,44,45,46,47,48,49,50] can be found in Supplementary Materials.
